# Left Atrium Thrombi Extending From Pulmonary Vein Thrombi Are Heterogeneous, Exhibit Calcifications, and Can Cause Acute Myocardial Infarction

**DOI:** 10.7759/cureus.78334

**Published:** 2025-02-01

**Authors:** Hidekazu Takeuchi

**Affiliations:** 1 Internal Medicine (Cardiology), Takeuchi Naika Clinic, Ogachi-Gun, JPN

**Keywords:** acute ischemic stroke (ais), acute myocardial infarction (ami), calcification, heterogeneous, left atrium thrombi, pulmonary vein thrombi

## Abstract

Previous studies have shown that thrombi, upon retrieval, sometimes exhibit calcification and endothelialization, indicating that they are old, and they can be classified as red clots, white clots, or mixed clots according to the degree of heterogeneity of the thrombus. These findings suggest that, prior to the occurrence of acute myocardial infarction (AMI) and acute ischemic stroke (AIS), such large and heterogeneous thrombi may already be present. Pulmonary vein thrombi (PVTs) are common in patients with age-related diseases. During lung infection, neutrophil extracellular traps (NETs) are generated and have the potential to form thrombi. We previously reported that warfarin and novel oral anticoagulants (NOACs) can partially resolve PVTs, indicating that PVTs are heterogeneous. However, it is unclear whether PVTs contain areas of calcification. In the present case, we describe a patient with left atrium (LA) thrombi extending from PVTs that contained calcifications according to cardiac computed tomography (CT) and transesophageal echocardiography (TEE). On these imaging modalities, the calcifications of the extended LA thrombi appeared as white areas and white areas with white shadows, respectively. Some thrombi, including calcifications, can be resolved with dabigatran, whereas others cannot, indicating that thrombi, including calcifications, are heterogeneous. The effects of microclots, including NETs released from PVTs, have been well studied, and NETs have been reported to be related to many diseases. Treatments for PVTs also produce beneficial effects against these diseases; however, the direct effects of PVTs on the heart and lungs are not well known.

## Introduction

In developed countries, acute myocardial infarction (AMI) ranks as the leading cause of death, and acute ischemic stroke (AIS) ranks as the second leading cause of death. Annually, AMI occurs in 3.8% of patients under 60 years of age and 9.5% of patients aged 60 years and over, and AIS affects approximately 800,000 patients in the United States. Among them, 600,000 patients experienced AIS for the first time [1]. AISs often result in death or severe disabilities and high social costs and burdens, costing approximately 56.5 billion dollars from 2018 to 2019 [1].

After confirming the diagnosis, a thrombectomy is performed for vessel occlusion recanalization to remove occluded thrombi mechanically. The characteristics of thrombi in patients with AMI or AIS can be determined by histological examination via microscopy with various dyes [2,3]. Previous thrombolysis studies have shown that such retrieved thrombi sometimes exhibit calcification [2,3] and endothelialization [2], indicating that these thrombi are chronic thrombi and present prior to the manifestation of AMI or AIS. Fresh thrombi do not exhibit calcification or endothelialization, and time is required to achieve calcification and endothelialization. These diseases can be prevented by treating preexisting thrombi, which very likely break off from larger thrombi in certain arteries, the pulmonary veins, the left ventricle, or the left atrium (LA).

The components of these thrombi mainly include platelets, erythrocytes, and fibrin; subsequently, the thrombi can be classified into three classes, red clots, white clots, and mixed clots, reflecting the degree of heterogeneity within the thrombi. Specifically, white clots, which mainly include fibrin and platelets, tend to be relatively stiff, whereas red clots, which mainly include erythrocytes, tend to be relatively flexible [4]. Red clots can be easily resolved by treatment. Taken together, these characteristics suggest that prior to the occurrence of AMI and AIS, relatively large, stable, heterogeneous thrombi may already exist in the body.

Pulmonary vein thrombi (PVTs) are common in patients with age-related diseases such as hypertension, dyslipidemia, angina pectoris, heart failure, and type 2 diabetes mellitus (T2DM) [5-9]; however, PVTs are not necessarily associated with particular symptoms. During lung infection, neutrophil extracellular traps (NETs) are generated to kill pathogens [10]; these structures have the ability to form thrombi that act as scaffolds in smaller veins to prevent the spread of pathogens to other organs [11]. These fine thrombi may subsequently gradually become enlarged and enter the larger pulmonary vein and then the LA [6].

We previously reported that warfarin and novel oral anticoagulants (NOACs) reduce thrombus size, meaning that NOACs partially resolve PVTs [5-9] according to findings from cardiac computed tomography (CT) and transesophageal echocardiography (TEE), the latter of which involves finding PVTs as white, gray, or black regions [7,8], reflecting their heterogeneity. However, it is unclear whether PVTs contain areas of calcification. If PVTs do not contain any calcification, it is difficult to say that PVTs can cause AMI or AIS because retrieved thrombi contain calcifications.

## Case presentation

A 66-year-old female with known long-standing hypertension and hyperlipidemia complained of dizziness over the past few years. The patient occasionally experienced brief episodes of dizziness without vomiting or ringing in her ears when she stood up. The patient was examined with transthoracic echocardiography (TTE) as a routine check-up to assess heart function. She had no symptoms of difficulty breathing, pyrexia, coughing, phlegm, tremor, or gait disturbance. The patient’s chest was clear on percussion and auscultation without lung crackles or wheezing. The heart-related findings on percussion and auscultation were within normal limits. Her body mass index (BMI) was 18.5 kg/m^2^, her weight did not decrease, her blood pressure was 132/78, and her pulse rate was 54 beats/min and regular. The patient was treated with medication for hypertension and hyperlipidemia; she had no history of thromboembolism, and no previous treatment with NOACs was reported. A chest X-ray revealed no pleural effusion or cardiomegaly. Electrocardiography revealed sinus rhythm with bradycardia, a normal axis, and no ST-segment or T-wave changes. The patient’s heart rate was 52 beats/min. The serum D-dimer level was 0.7 μg/ml (normal range: <1.0 μg/ml), the activity of protein S was 81% (normal range: 60-127%), and the activity of protein C was more than 151% (normal range: 64-135%). The homosistein level was 5.7 nmol/mL (normal range: 5-15 nmol/mL).

**Figure 1:**
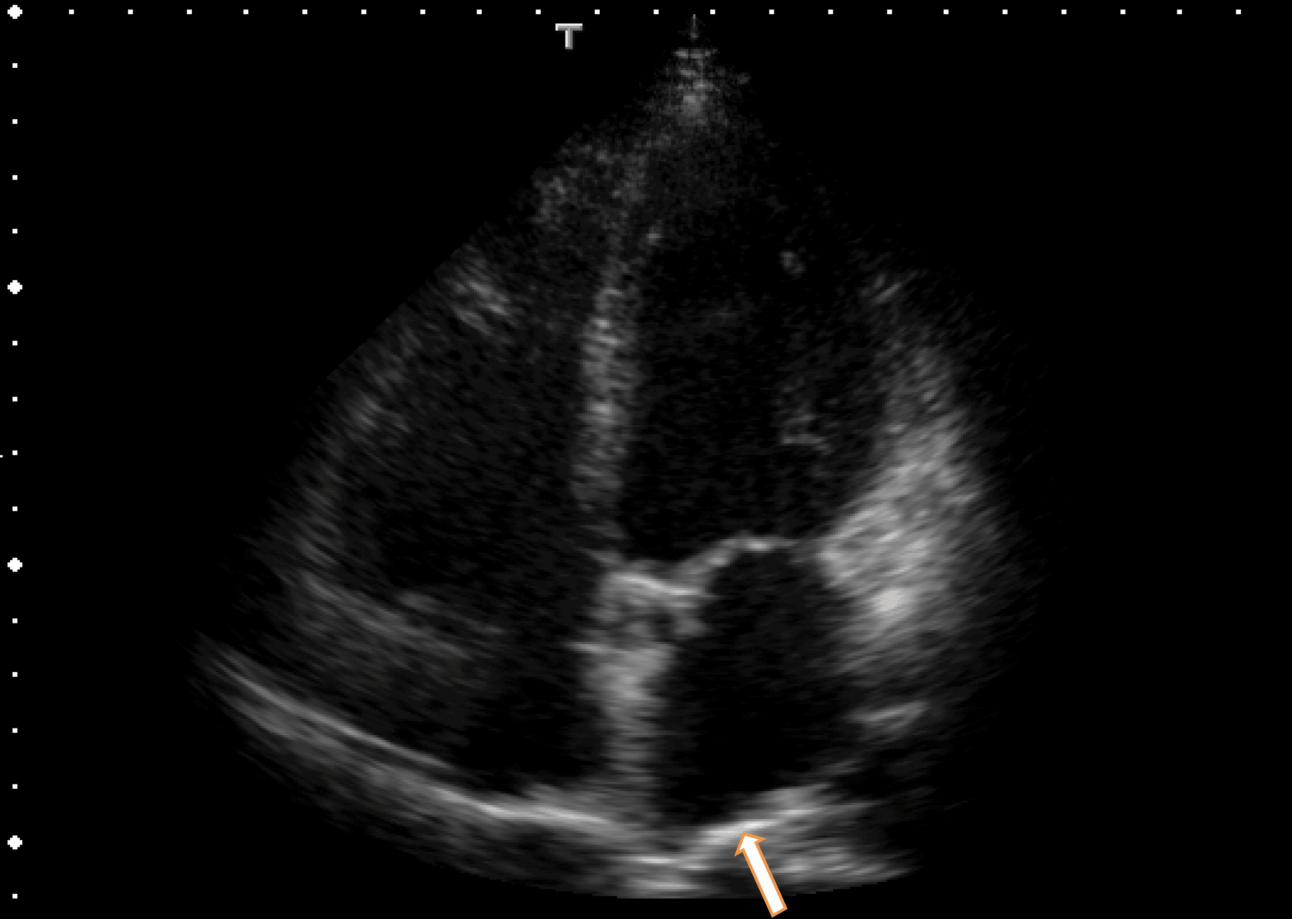
Transthoracic echocardiography (TTE) images showing left atrium (LA) thrombi TTE images showing a hyperechoic region in the LA around the base of the right upper pulmonary vein (RUPV) (arrow).

Surprisingly, TTE revealed a hyperechoic region around the exit of the right upper pulmonary vein in the LA (Fig. 1, Video 1), indicating that LA thrombi extended from PVTs, which can cause dizziness by many mechanisms. PVTs and extended LA thrombi can release micloclots, which can occlude the microartery of the brain; thus, dizziness may occur.

**Video 1:** Video images from transthoracic echocardiography (TTE) TTE images revealed red flow from the right upper pulmonary vein (RUPV) and a hyperechoic region in the left atrium (LA) around the ostia of the RUPV.

**Figure 2:**
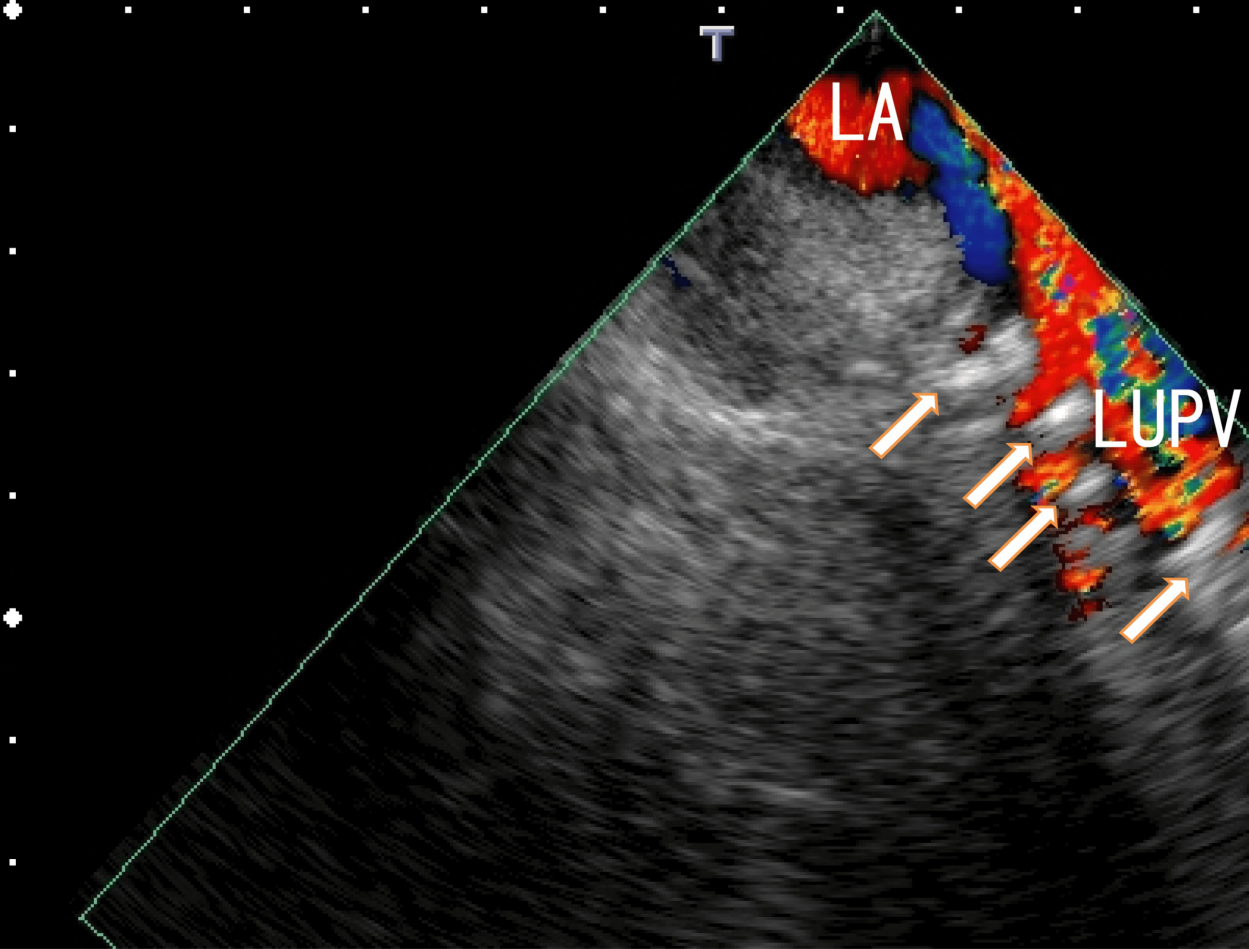
Transesophageal echocardiography (TEE) images showing thrombi TEE images showing thrombi in the left upper pulmonary vein (LUPV) (arrows). The thrombi are attached to the wall of the LUPV and present as several hyperechoic regions. The average size of the hyperechoic regions measured approximately 2 mm × 5 mm and were arranged perpendicularly to the direction of the LUPV. LA: left atrium, LUPV: left upper pulmonary vein

**Figure 3:**
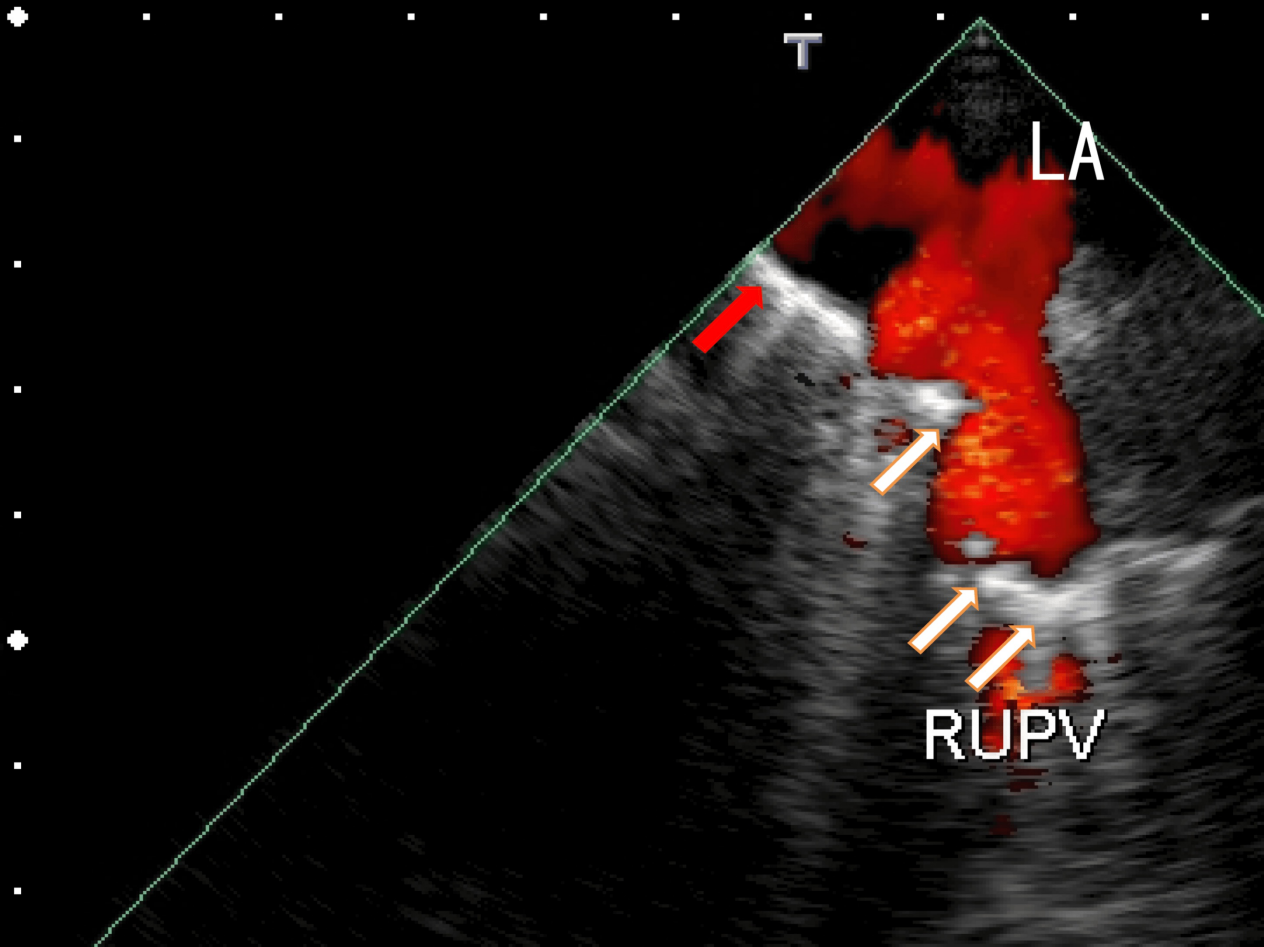
TEE images showing thrombi TEE images showing thrombi in the RUPV (white arrows) and the LA (red arrow). The LA thrombi extend from the right lower pulmonary vein (RLPV) thrombi and appear as a white line parallel to the direction of the RLPV. The size of the hyperechoic region is approximately 3 mm × 15 mm. The other several thrombi are attached to the wall of the RUPV and present as hyperechoic regions. These hyperechoic regions measure approximately 3 mm × 7 mm and are perpendicular to the direction of the RUPV. Some parts of the thrombi have white shadows, indicating areas of calcification. Some of the white areas are surrounded by dark areas that connect to other thrombi and are attached to the LA wall. TEE: transesophageal echocardiography, LA: left atrium, RUPV: right upper pulmonary vein

For further investigation of this hyperechoic region, TEE was performed. TEE revealed several thrombi manifesting as white areas with white shadows in the left upper pulmonary vein (LUPV) (Fig. 2, Video 2) and the right upper pulmonary vein (RUPV) (Fig. 3 and Video 3). White areas with white shadows are suggestive of thrombus composition and calcification. In addition, LA thrombi extended from thrombi in the right lower pulmonary vein (RLPV) (Fig. 3, Video 3). Dark areas surrounding the white areas of the thrombi were also identified, indicating that these thrombi were heterogeneous. These thrombi release some clots from micro- to large thrombi; large clots can cause AMI or AIS, and microclots can prevent normal cell function via the occlusion of microvessels. These dark areas surrounding the white areas of the thrombi may suggest the presence of different thrombus stages.

**Video 2:** Video images from transesophageal echocardiography (TEE) TEE images revealed several thrombi in the left upper pulmonary vein (LUPV) depicted as small white masses. Some thrombi contain white shadows and move with the heartbeat. Some dark areas can be detected around the white areas. Red indicates blood flow in the LA from the LUPV. LA: left atrium, LUPV: left upper pulmonary vein

**Video 3:** Video images from TEE Transesophageal echocardiography (TEE) images revealed LA thrombi extending from right lower pulmonary vein (RLPV) thrombi, which appeared as a line-like white mass around the exit of the right middle pulmonary vein (RMPV), and flow from the RMPV could not be detected. The line-like thrombus sometimes contains white shadows and does not move with the heartbeat; however, the thrombus moves with breathing. The white mass is surrounded by dark areas. TEE images revealed another line-like white mass around the exit of the right upper pulmonary vein (RUPV). The right half of the thrombus does not move with the heartbeat; however, the left half of the thrombus moves with the heartbeat. The left ends of the extended LA thrombi are connected to the anterior wall of the LA, and calcified thrombi around the exit of the RUPV have relatively strikingly dark areas. Red indicates blood flow from the RUPV.

**Figure 4:**
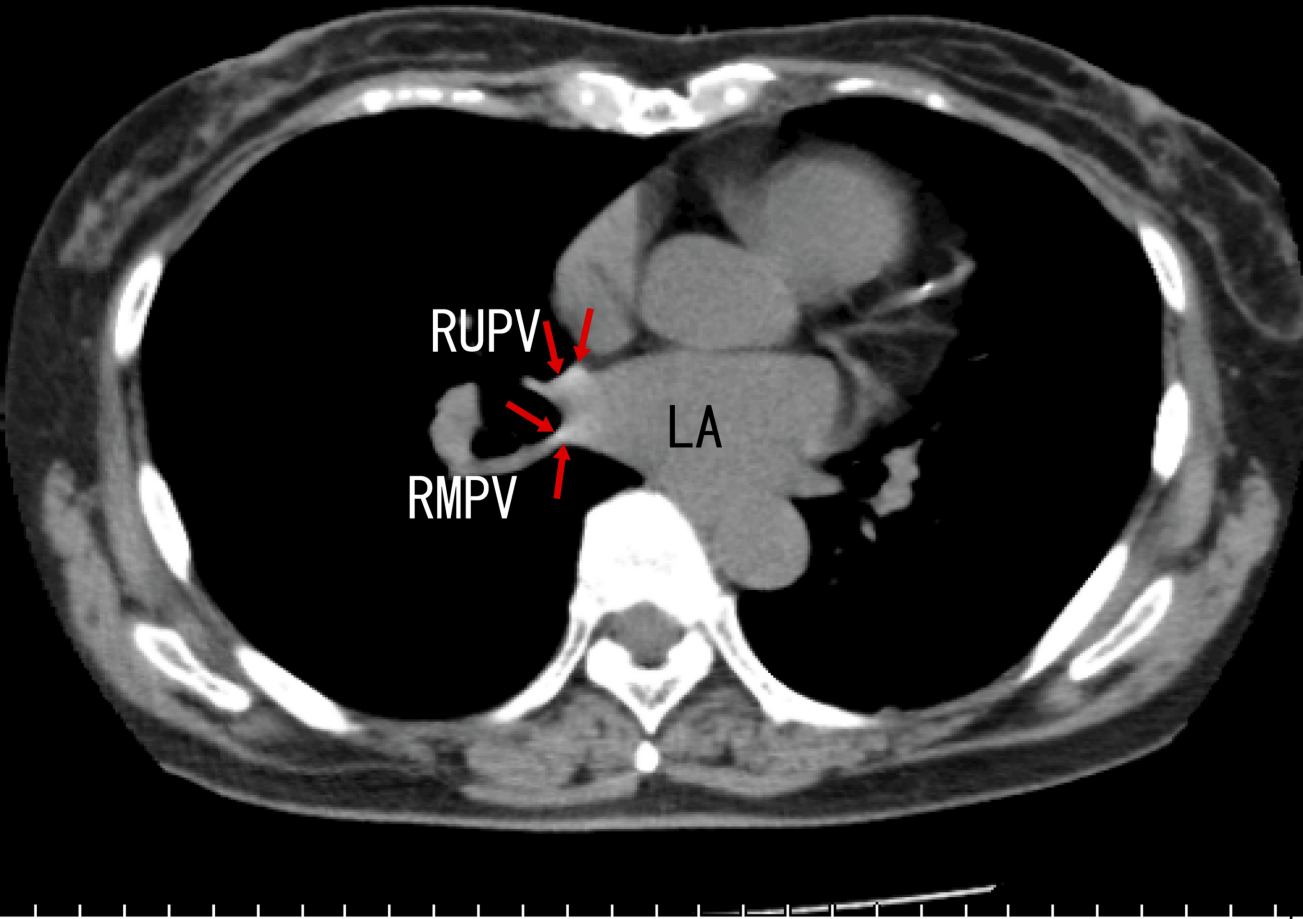
Axial plain computed tomography (CT) images Axial images depict calcification in the RUPV and right middle pulmopnary vein (RMPV) as white areas (arrows). LA: left atrium, RMPV: right middle pulmonary vein, RUPV: right upper pulmonary vein

**Figure 5:**
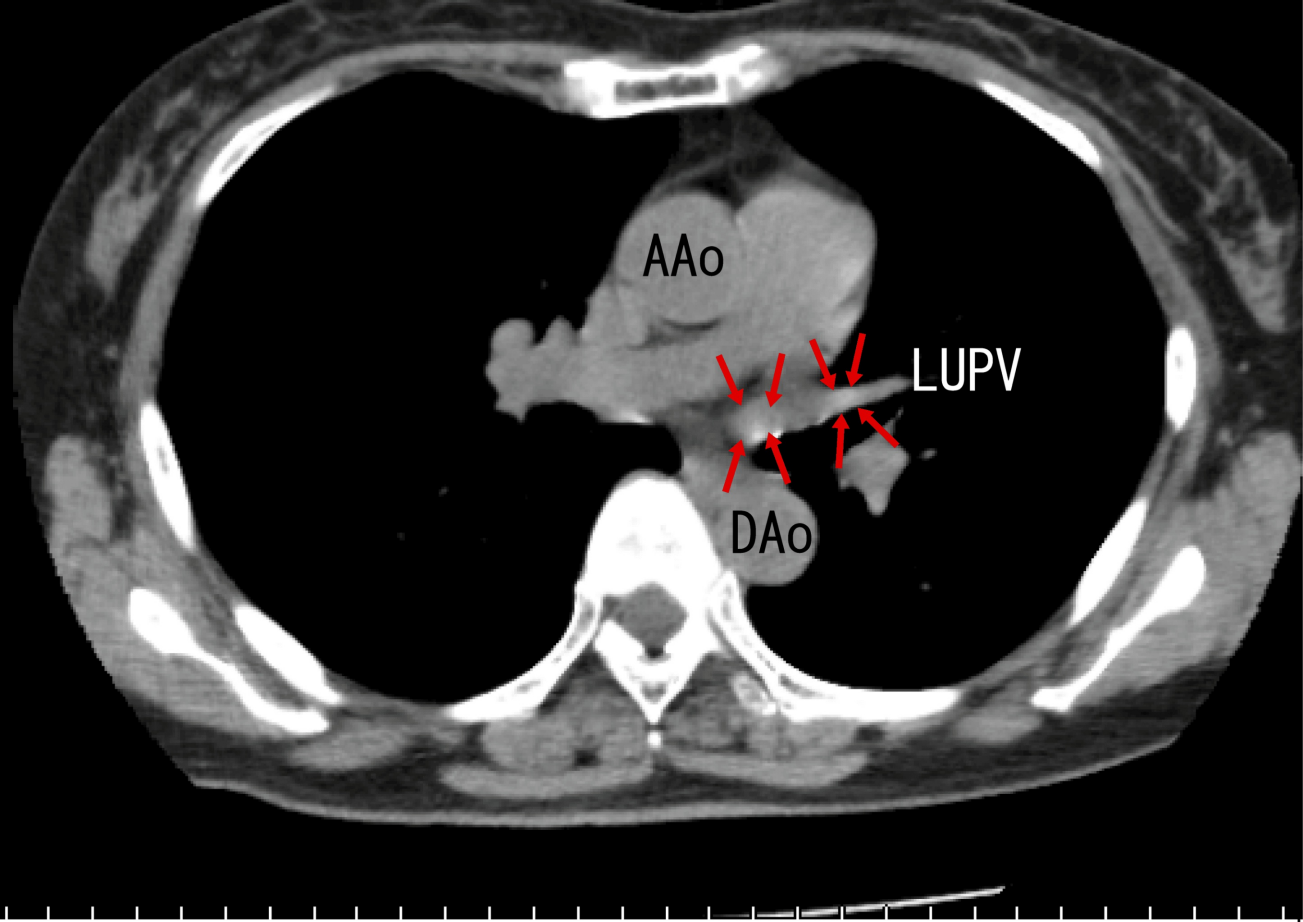
Axial plain CT images Axial images depict calcifications in the LUPV as two white areas (arrows). AAo: ascending aorta, DAo: descending aorta, LUPV: left upper pulmonary vein

Finally, plain chest computed tomography (CT) revealed calcification around the exit of the RUPV and the right middle pulmonary vein (RMPV) (Fig. 4) and in the LUPV (Fig. 5) as white areas. The presence of these white areas suggested that there were calcifications in these regions.

**Figure 6:**
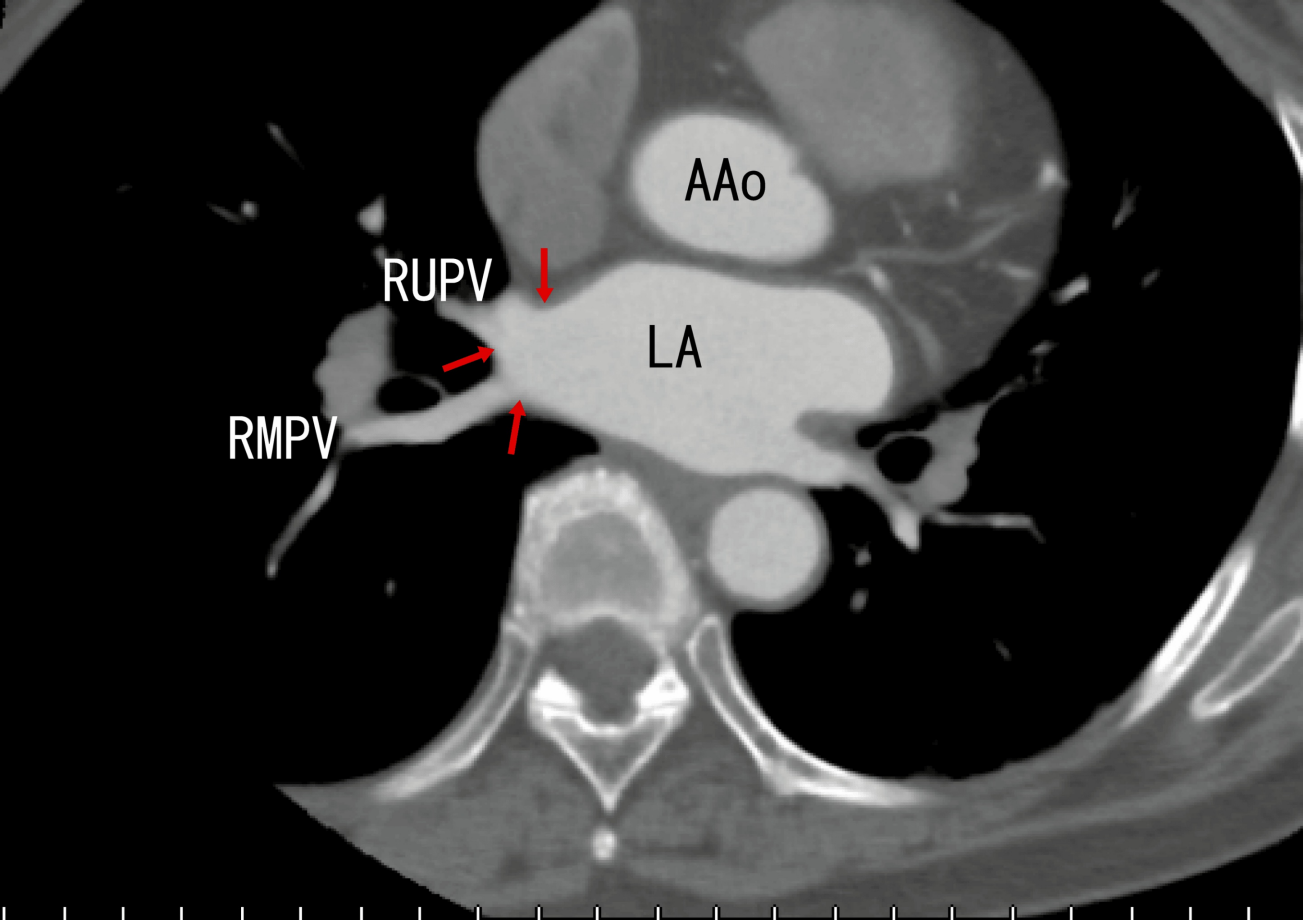
Axial enhanced CT images The axial images revealed no thrombi around the exit of the RMPV or RUPV (arrows). AAo: ascending aorta, LA: left atrium, RMPV: right middle pulmonary vein, RUPV: right upper pulmonary vein

**Figure 7:**
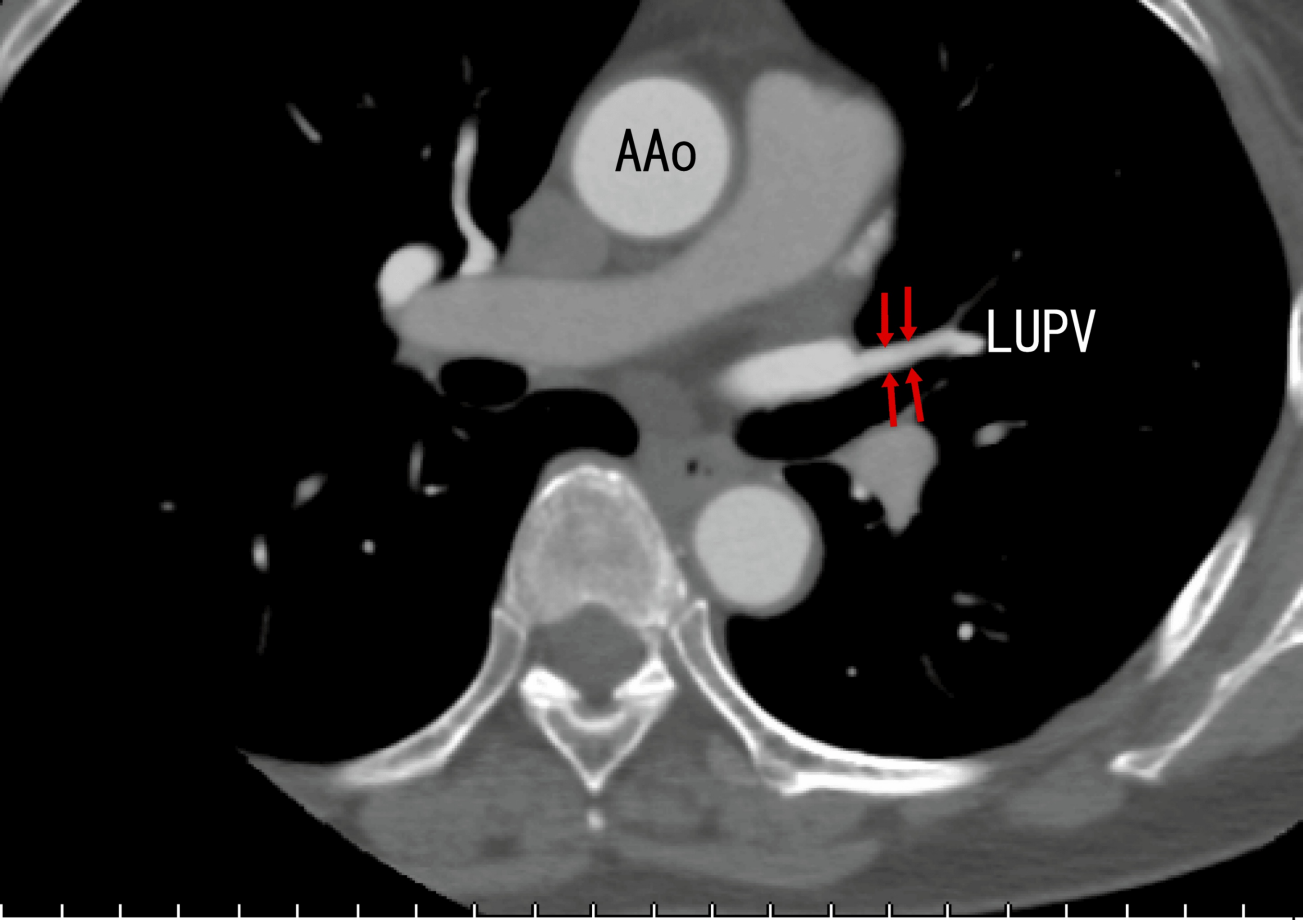
Axial-enhanced CT images Axial images depict no thrombi in the LUPV (arrows). AAo: ascending aorta, LUPV: left upper pulmonary vein

However, enhanced CT revealed no thrombi around the exit of the RUPV or RMPV (Fig. 6) or in the LUPV (Fig. 7). These images were obtained before dabigatran treatment. Reportedly, PVTs and extended LA thrombi from PVTs are often not detectable using enhanced CT [8,9], which is a basic and important trait and may be the main cause of their underestimation.

**Figure 8:**
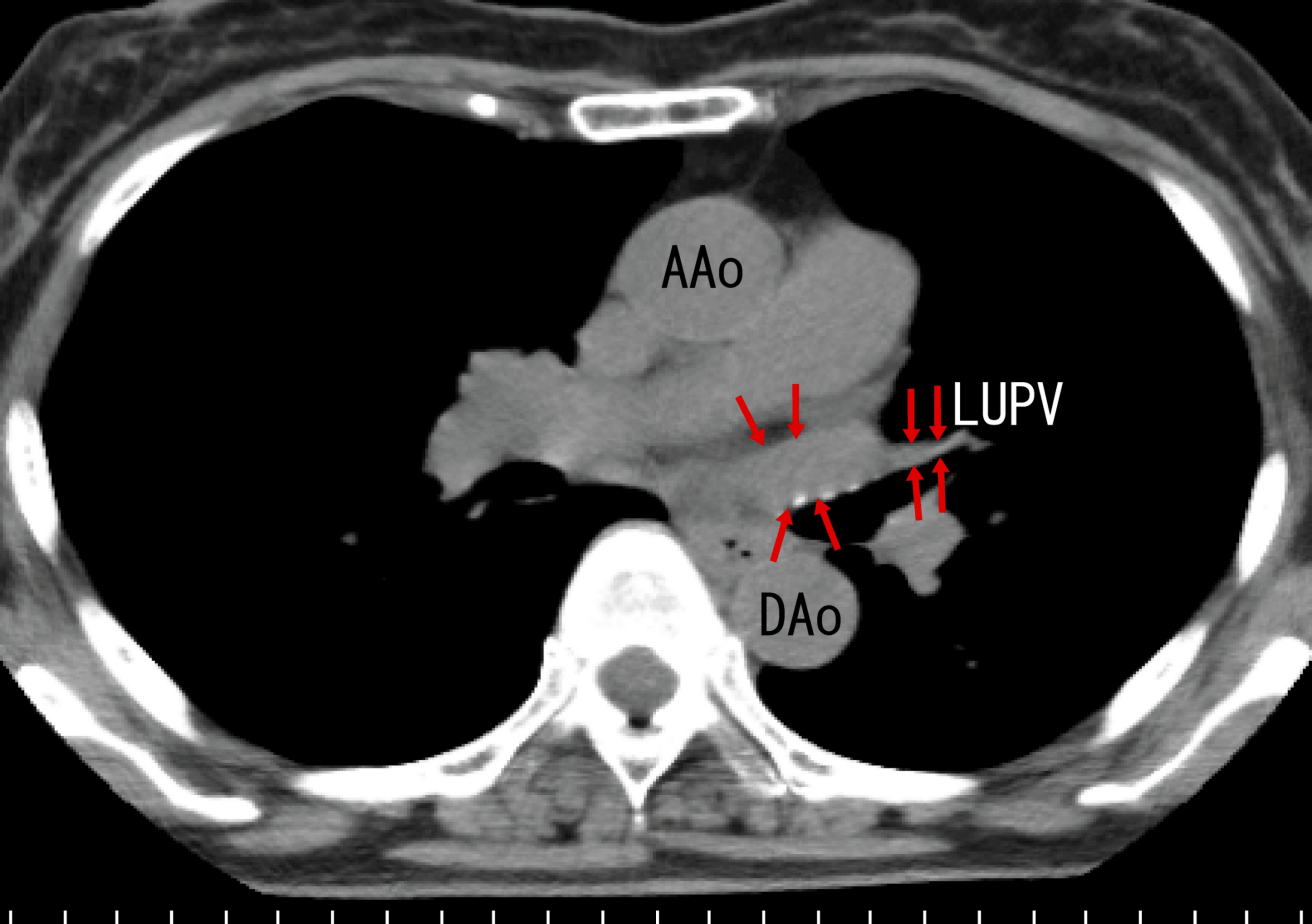
Axial plane CT images Axial images revealed no calcified thrombi in the LUPV as white areas (arrows); however, they revealed the cartilarge of the left bronchus as three white spots. AAo: ascending aorta, DAo: descending aorta, LUPV: left upper pulmonary vein

**Figure 9:**
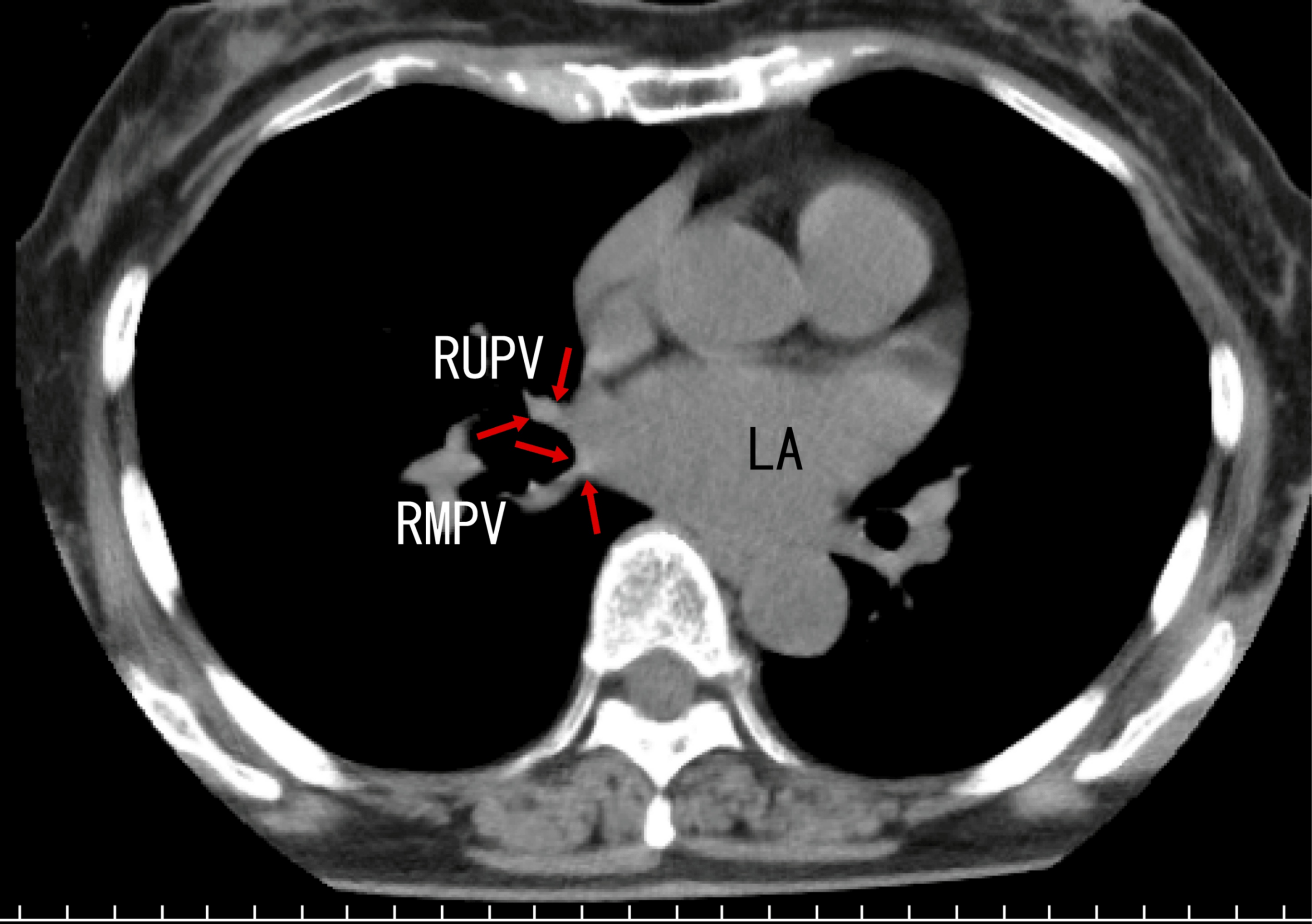
After three months of dabigatran treatment, axial plain CT images After three months of dabigatran treatment, axial images revealed calcification around the exit of the RUPV and RMPV as white areas (arrows). Compared with Fig. 4, the white areas decreased to some extent, especially around the exit of the RUPV. LA: left atrium, RMPV: right middle pulmonary vein, RUPV: right upper pulmonary vein

We treated the patient with dabigatran (110 mg: twice daily) for three months. We used dabigatran because, on the basis of our experience, we believe that dabigatran could resolve PVTs. After three months of dabigatran treatment, these thrombi, including calcifications in the LUPV disappeared (Fig. 8); however, the number of thrombi, including calcifications around the exits of the RUPV and RMPV, decreased (Fig. 9), indicating that these thrombi might be heterogeneous.

**Figure 10:**
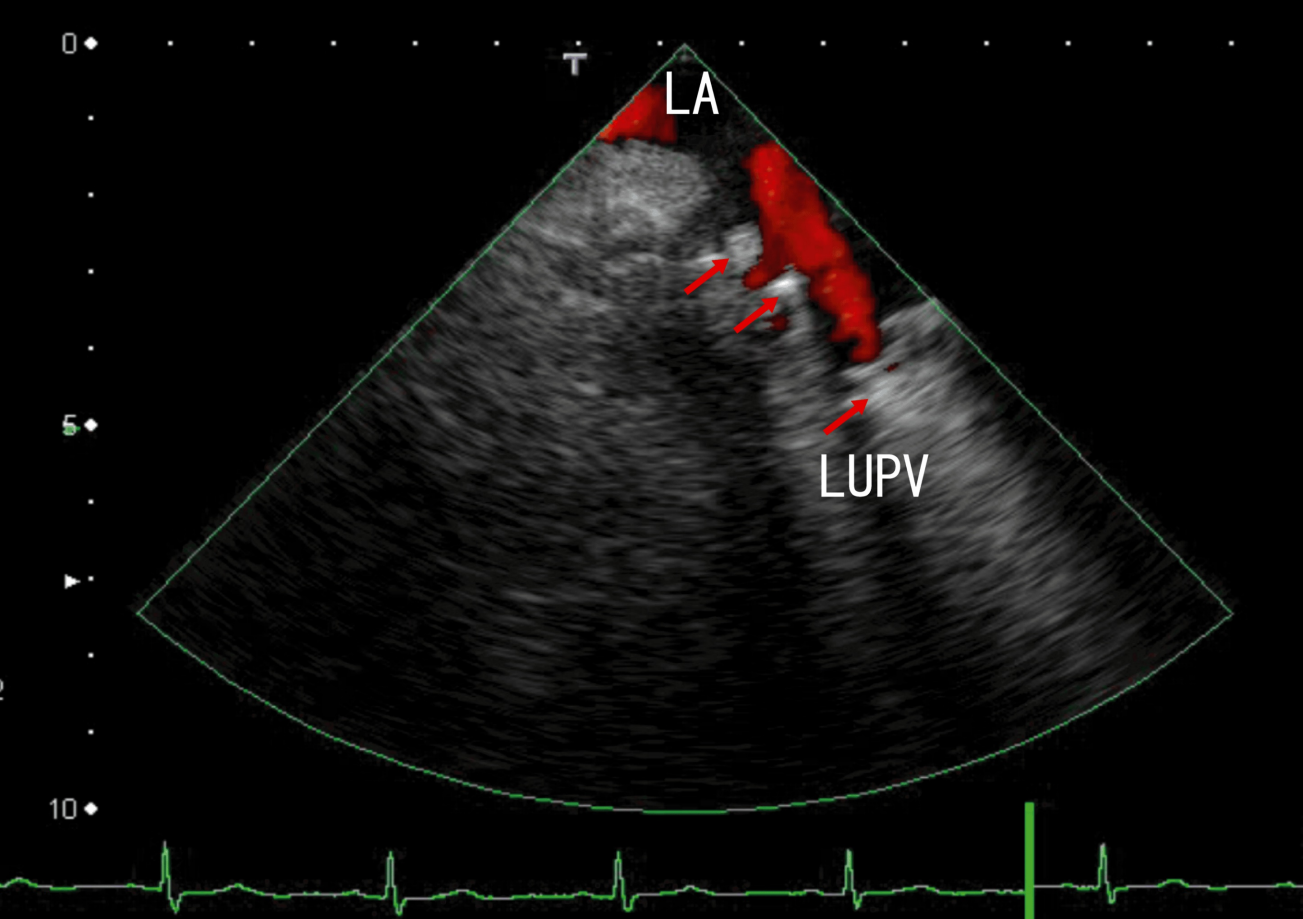
TEE images showing thrombi after three months of dabigatran treatment After three months of dabigatran treatment, TEE images revealed thrombi in the LUPV (arrows). The thrombi are attached to the wall of the LUPV and present as several hyperechoic regions. The average size of the hyperechoic regions measured approximately 2 mm × 4 mm and were arranged perpendicularly to the direction of the LUPV. LA: left atrium, LUPV: left upper pulmonary vein

TEE revealed that the white thrombi around the exit of the LUPV were not clearly resolved; however, decreased dark thrombi on the wall of the LUPV were detected (Fig. 10, Video 4).

**Video 4:** Video images from TEE after three months of dabigatran treatment After three months of dabigatran treatment, TEE revealed several thrombi in the LUPV depicted as small white masses. Some thrombi contain white shadows and move with the heartbeat. Some dark areas in the LUPV decreased, and the red areas in the LUPV area broadened. Red indicates blood flow in the LA from the LUPV area.

**Figure 11:**
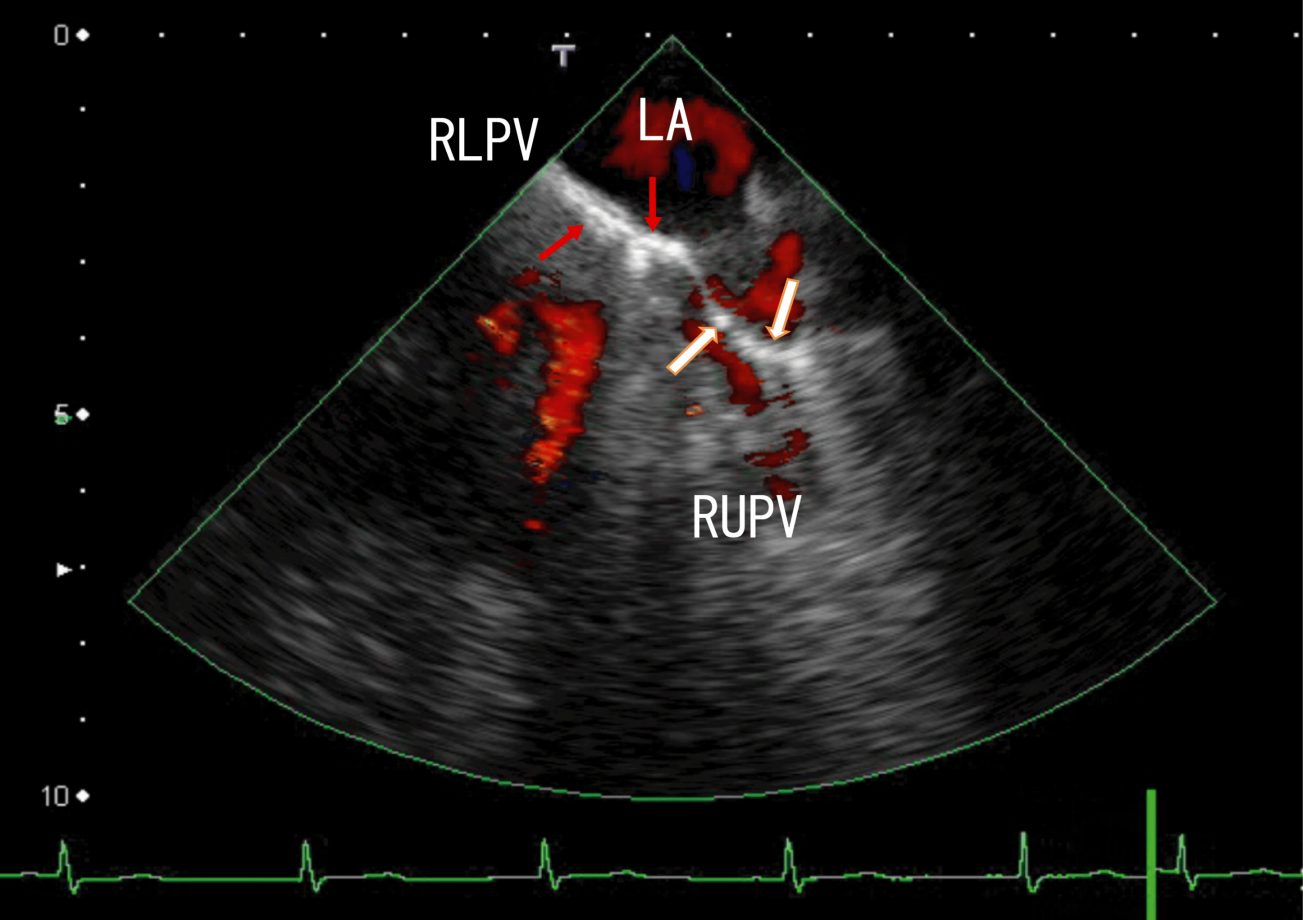
TEE images showing thrombi after three months of dabigatran treatment After three months of dabigatran treatment, TEE images revealed thrombi in the RUPV (white arrows) and the LA (red arrow). The LA thrombi extend from the right lower pulmonary vein (RLPV) thrombi and appear as a white line parallel to the direction of the RLPV. The size of the hyperechoic region is approximately 3 mm × 20 mm. The other thrombi are attached to the wall of the RUPV and present as hyperechoic regions. These hyperechoic regions measure approximately 2 mm × 12 mm and are perpendicular to the direction of the RUPV. Some parts of the thrombi have white shadows, indicating areas of calcification. Some of the white areas are surrounded by dark areas that connect to other thrombi and are attached to the LA wall. LA: left atrium, RLPV: right lower pulmonary vein, RUPV: right upper pulmonary vein

The white thrombi around the exit of the RUPV and RMPV did not clearly change; however, the number of thrombi situated between the line-like white mass and the LA wall decreased; therefore, blood flow from the RMPV was detected (Fig. 11, Video 5).

**Video 5:** Video images from TEE after three months of dabigatran treatment After three months of dabigatran treatment, TEE images revealed LA thrombi extending from RLPV thrombi, which appeared as a line-like white mass around the exit of the RMPV, and the flow from the RMPV could be detected, indicating resolution of the thrombi located between the line-like white mass and the LA wall. The line-like thrombus sometimes contains white shadows and does not move with the heartbeat; however, the thrombus moves with breathing. TEE images revealed another line-like white mass around the exit of the RUPV, which moves with the heartbeat. The left ends of the extended LA thrombi are connected to the anterior wall of the LA, and calcified thrombi surround the exit of the RUPV. Red indicates blood flow from the RUPV and RMPV. TEE: transesophageal echocardiography, LA: left atrium, RLPV: right lower pulmonary vein, RMPV: right middle pulmonary vein, RUPV: right upper pulmonary vein

After three months, we thought that dabigatran did not work well and that rivaroxaban could resolve those thrombi more, so we treated the patient with rivaroxaban (10 mg: once daily). However, we did not detect any clear changes after the treatment using rivaroxaban. During these treatments, the patient did not feel dizzy.

## Discussion

To our knowledge, this is the first report describing PVTs containing calcifications. Calcifications are often observed within retrieved thrombi, which indicates that PVTs are a source of the thrombi that cause AMI or AIS. This is an important finding; since PVTs can be resolved with warfarin or NOACs, it should be possible to decrease the occurrence of AMI or AIS to a certain extent. Additionally, this is the first study to report that PVTs are heterogeneous, as they are composed of white and dark areas on TEE. The retrieved thrombi can be classified as red clots, mixed clots, or white clots, again reflecting their heterogeneity, which was the case for the PVTs identified in this study. Additionally, both retrieved thrombi and PVTs have calcifications, which indicates that they are old.

Recently, several studies have reported that NETs are present in retrieved thrombi [12], form thrombi [11] and stabilize thrombi [12]. Thus, NETs may promote PVT enlargement. Micro-PVTs may be produced and stabilized by NETs. Consequently, these thrombi slowly enlarge and sometimes reach the LA [6]. This possibility was first discussed in this study. The LA thrombi extended from the RLPV thrombi parallel to this vein; this extension appeared as a stiff line on imaging and consisted of numerous white spots. However, the white thrombi in the RUPV and the LUPV extended in a direction perpendicular to the pulmonary vein. TEE images revealed that these white thrombi included some areas that produced white shadows, suggesting areas of calcification. However, some white regions did not have shadows, which may represent fiber films [13] and delay thrombolysis. Large areas of calcification might form in these thrombi with fibrin films, and this possibility was first discussed in this study.

Plane CT images revealed that the white areas around the exit of the RUPV and RMPV did not clearly change despite dabigatran treatment; however, the white areas in the LUPV disappeared. These differences may be due to the nature of the fibrin film, indicating that fibrin films are not homogeneous. Plane CT images sometimes revealed white areas around the exit of the RUPV and RMPV (data not shown); however, white areas around the exit of the LUPV were not detected often. There were white areas around the exit of the RMPV, which reflected the existence of white extended LA thrombi with white shadows from the RLPV thrombi. However, the left lower pulmonary vein (LLPV) thrombi did not extend white LA thrombi, as in this case. The white thrombi around the exit of the RUPV and RMPV differ from the white thrombi in the LUPV, meaning that they are heterogeneous, which was discussed in this study. Although enhanced CT did not directly detect these thrombi in the LA or pulmonary veins, these thrombi should have been detected as lacking enhancement; however, the surfaces of the LA and pulmonary vein were smooth and had no lack of enhancement. Extended LA thrombi from PVTs often cannot be detected by enhanced CT, which is a surprising characteristic of those thrombi. The surfaces of those thrombi may be covered by the endothelium, similar to retrieved thrombi [2], which have the potential to affect thrombus detection by enhanced CT; however, the mechanisms underlying this phenomenon are unclear.

PVTs can release not only large thrombi that cause AMI or AIS but also microclots, including NETs. NETs are reportedly related to many diseases, such as atherosclerosis [14], heart failure [15], osteoporosis [16], inflammatory bowel disease [16], T2DM [17], and cancer [18]. PVTs can be partially decreased with warfarin, NOACs, and heparin, which also have some beneficial effects on the abovementioned diseases. In particular, heparin and warfarin treatment have very beneficial effects on patients with mild to moderate T2DM, greatly decreasing the use of DM medications [5,7] and sometimes eliminating their need completely. Heparin has been reported to destroy NETs by breaking histones, so heparin and warfarin treatment can resolve micloclots and restore microvessel blood flow. Thus, insulin can restore normal function, so DM medication is not needed. Patients with severe T2DM were not affected by heparin or warfarin treatment because the insulin secretion system itself was disrupted. Atherosclerosis, heart failure, osteoporosis, inflammatory bowel disease, and cancer might be curable using heparin and warfarin treatment.

The direct effects of PVTs and extended LA thrombi from PVTs on the heart and lungs are unclear. We reported that extended LA thrombi from PVTs attach to the anterior wall of the LA; then, left atrium diverticula (LADs) exist near the attachment areas. We reported that the LAD transformed into a circular LAD [5]. The retrieved thrombi contained CD34-positive cells [2]. The extended LA thrombi might be associated with LAD formation and transformation via CD34-positive cells. Leukocytes on extended LA thrombi may be associated with leukocytes on the LA wall, which may be associated with heart failure. In video 2, several small thrombi containing white shadows move with the heartbeat; however, in video 3, the line-like large thrombus containing white shadows moves with not the heartbeat but the breathing. Many thrombi exist between the line-like thrombus and the LA wall, and those thrombi attach to the LA wall, which is located around the exit of the RMPV. The LA wall may lose normal contraction ability there, so line-like thrombus does not move with the heartbeat, which may be a start of heart failure. There is a possibility that extended LA thrombi are associated with the production of end-QRS notches and early repolarization (ER), which are restored via NOACs [19]. It is unclear whether pulmonary hypertension can be caused by PVTs. To clarify these relationships, additional studies, including clinical trials using warfarin NOACs and heparin, imaging studies, and molecular research, are needed.

Cardiogenic embolism is often used clinically, and left atrial appendage (LAA) thrombi are considered a source of cardiogenic embolism in patients with atrial fibrillation (AF). However, AIS occurs in patients without AF, and at present, the source of cardiogenic embolism in patients without AF is unclear. PVTs and extended LA thrombi from PVTs can be sources of cardiogenic embolism. Previous studies have shown that retrieved thrombi exhibit calcification; however, LAA thrombi have not been reported to exhibit calcification, and our data indicate that LAA thrombi do not exhibit calcification, as estimated via TEE and plane CT (data not shown). The existence of calcification suggests that a source of cardiogenic embolism is likely PVTs rather than LAA thrombi. Treating PVTs in the early stage can decrease the occurrence of AMI and AIS because the number of PVTs can be decreased by treatment with warfarin and NOACs; however, these treatments have side effects. Exercise can promote the secretion of tissue plasminogen activator (t-PA) [20], which can decrease NETs, resolve occluded thrombi and resolve PVTs. Young people tend to exercise more than elderly people, at least by walking, and as a result, the homeostasis of PVTs in young people tends to be well balanced, and PVTs are generally stable. However, elderly people tend to move their bodies less, leading to a decreased production of tPA and a subsequent disruption of homeostasis, leading to PVTs becoming larger and sometimes unstable, leading to AMI and AIS. Therefore, we may need to exercise as much as possible to avoid these risks, even when we are older. These are first discussed in this study. More clinical studies are needed to clarify these relationships.

## Conclusions

Calcifications of LUPV and RUPV thrombi and LA thrombi extending from RLPV thrombi were demonstrated via TEE and plain CT. The calcifications were depicted as white areas on plain CT and as white areas with white shadows on TEE. Those thrombi were heterogeneous and included calcifications, which coincide with the characteristics of retrieved thrombi taken from patients with AMI or AIS. After three months of dabigatran treatment, calcifications in the thrombi around the RUPV and RMPV were present despite the disappearance of calcifications in the thrombi around the LUPV, as evaluated via plane CT. After three months of dabigatran treatment, TEE revealed calcifications as white areas with white shadows; however, the number of dark thrombi around the white areas decreased.
